# Hemimycalins C–E; Cytotoxic and Antimicrobial Alkaloids with Hydantoin and 2-Iminoimidazolidin-4-one Backbones from the Red Sea Marine Sponge *Hemimycale* sp.

**DOI:** 10.3390/md19120691

**Published:** 2021-12-02

**Authors:** Lamiaa A. Shaala, Diaa T. A. Youssef

**Affiliations:** 1 Natural Products Unit, King Fahd Medical Research Center, King Abdulaziz University, Jeddah 21589, Saudi Arabia; 2 Department of Medical Laboratory Sciences, Faculty of Applied Medical Sciences, King Abdulaziz University, Jeddah 21589, Saudi Arabia; 3 Suez Canal University Hospital, Suez Canal University, Ismailia 41522, Egypt; 4 Department of Natural Products, Faculty of Pharmacy, King Abdulaziz University, Jeddah 21589, Saudi Arabia; 5 Department of Pharmacognosy, Faculty of Pharmacy, Suez Canal University, Ismailia 41522, Egypt

**Keywords:** Red Sea sponge, *Hemimycale* sp., marine alkaloids, hydantoin and 2-iminoimidazolidin-4-one backbones, hemimycalins C–E, cytotoxicity, antimicrobial activity

## Abstract

In the course of our continuing efforts to identify bioactive secondary metabolites from Red Sea marine sponges, we have investigated the sponge *Hemimycale* sp. The cytotoxic fraction of the organic extract of the sponge afforded three new compounds, hemimycalins C–E (**1**–**3**). Their structural assignments were obtained via analyses of their one- and two-dimensional NMR spectra and HRESI mass spectrometry. Hemimycalin C was found to differ from the reported hydantoin compounds in the configuration of the olefinic moiety at C-5–C-6, while hemimycalins D and E were found to contain an 2-iminoimidazolidin-4-one moiety instead of the hydantoin moiety in previously reported compounds from the sponge. Hemimycalins C–E showed significant antimicrobial activity against *Escherichia coli* and *Candida albicans* and cytotoxic effects against colorectal carcinoma (HCT 116) and the triple-negative breast cancer (MDA-MB-231) cells.

## 1. Introduction

The marine environment has played an essential role in the discovery of compelling secondary metabolites with fascinating antitumor, immunomodulatory, analgesic, anti-inflammatory, anti-allergic, antimicrobial, and antiviral effects [1,2]. Since 1963, more than 30,000 new chemical entities have been identified from marine organisms, including macro- and micro-organisms [3]. Secondary metabolites obtained from marine invertebrates have received great attention from pharmacologists and chemists due to their remarkable chemical diversity and biological activities [4,5,6]. The fact that 14 marine-derived approved drugs and another 23 drug leads in different phases (I–III) of clinical trials [7], mostly from marine invertebrates [7], clearly indicates the role of marine invertebrates as a vigorous source for the drug-discovery process [7]. Sponges belonging to the genus *Hemimycale* are excellent producers of alkaloids with both guanidine [8,9] and hydantoin backbones [10,11]. Ptilomycalin A, with its exceptional polycyclic guanidine backbone linked with a ω-hydroxyhexadecanoyl-spermidine moiety via an ester linkage, has displayed notable antimicrobial and antiviral activities [8,9].

The skeletal muscle relaxant dantrolene and the anticonvulsive drugs phenytoin, norantoin, mephenythoin, ethotoin, methetoin, and fosphenytoin are hydantoin-derived compounds [11,12]. Similarly, 5-substituted hydantoins (5,5-dithienylhydantoin, 5,5-dipyridylhydantoin, dithiohydantoins, thiohydantoin, and spirothiohydantoin) have anticonvulsive activity [13,14]. Other significant activities for hydantoin derivatives include antimicrobial (nitrofurantoin), antiarrhythmic (azimilide), and nonsteroidal antiandrogens (nilutamide) activities. Allantoin is used as an antacid, antipsoriatic, keratolytic, and astringent, as well as in wound remedy [12]. Additionally, antiviral, antidepressant, and antithrombotic and enzyme inhibition are additional pharmacological properties of hydantoins [15]. Finally, the herbicidal effects of spirohydantoin and thioxohydantocidin, as well as the fungicidal properties of clodantoin, are attributed to the hydantoin backbone in their structures [16,17]. Recently, the in vitro anti-growth and anti-invasive effects of (*Z*)-5-(4-hydroxybenzylidene)imidazolidine-2,4-dione and its analogue (*Z*)-5-(4-(ethylthio)benzylidene)-hydantoin against PC-3M prostate cancer were reported [18]. The compounds reduced the growth of orthotopic tumors and repressed the formation of tumor micrometastases in distant organs without apparent cytotoxic effects at the test doses [18].

As a continuation of our work to uncover biologically active alkaloids from marine organisms [19,20,21,22], the cytotoxic fractions of a methanolic extract of the sponge *Hemimycale* species were investigated. Three new alkaloids, hemimycalins C–E (**1**–**3**) with hydantoin and 2-iminoimidazolidin-4-one backbones, were obtained from the active fractions of the extract, and their structures were characterized. Here, we report on the structural determination and the antimicrobial and cytotoxic activities of the compounds.

## 2. Results and Discussion

**Figure 1:**
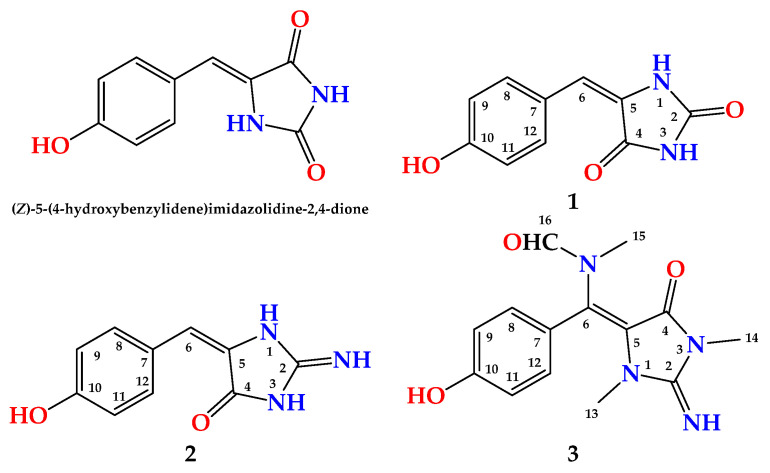
Structures of **1**–**3**.

**Figure 2:**
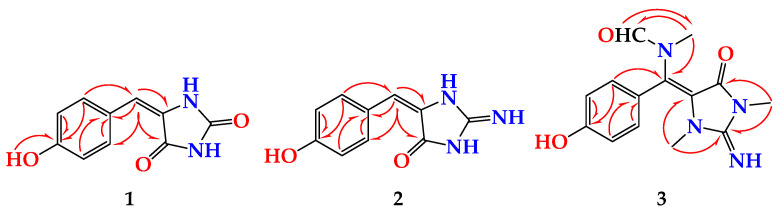
Key HMBC correlations in **1**–**3**.

Compound **1** (Figure 1) was obtained as a yellow powder. The molecular formula was C_10_H_8_N_2_O_3_, and it was obtained from the (+)-HRESIMS peak at *m/z* 205.0609 [M + H]^+^. The interpretation of its NMR spectral data including ^1^H (Figures S1 and S2), ^13^C (Figure S3), DEPT (Figure S4), HSQC (Figure S5) and HMBC (Figure S6) supported the structure of the compound. The ^1^H NMR spectra showed two parts: a benzene ring and an imidazolidine-2,4-dione (hydantoin) part connected together via a vinylic carbon (C-6) (Figure 1). The HMBC cross peaks from H-6 (δ_H_ 6.23) to C-4 (δ_C_ 163.6) and C-5 (δ_C_ 127.0) and from H-8 (δ_H_ 7.82) and H-12 ((δ_H_ 7.82) to C-6 (δ_C_ 116.8) supported the connection of the fragments of **1** through the vinylic C-6 (Table 1 and Figure 2). The ^1^H and ^13^C NMR signals of **1** were found to be similar to those of (*Z*)-5-(4-hydroxybenzylidene)imidazolidine-2,4-dione [10] with differences in the chemical shifts of some ^1^H and ^13^C NMR signals (Table 2). In a comparison of the NMR data of (*Z*)-5-(4-hydroxybenzylidene)imidazolidine-2,4-dione [10] with those of **1**, a significant downfield shift of C-6 (Δδ_C_ = + 7.6 ppm) was observed in **1**, suggesting a different configuration of Δ^5,6^ in **1**. Additional ^13^C NMR chemical shift variations were observed in the imidazolidine-2,4-dione moiety (C-2, C-4 and C-5) ranging from −2.1 to +1.7 ppm (Table 2).

It is well known that H-6 possesses a higher chemical shift value in *Z*-configured double bonds than in the *E*-configured ones [23,24]. Additionally, the ^13^C chemical shift of C-6 is more highly downfield in compounds with the *E* configuration than those with the Z configuration [25]. This effect could be a result of both anisotropic and diamagnetic effects on H-6 by the adjacent carbonyl group (C-4) [23]. In addition, significant downfield shifts (+0.36 ppm) for the signals of H-8 and H-12 in **1** when compared to those reported for (*Z*)-5-(4-hydroxybenzylidene)imidazolidine-2,4-dione [10] were noticed. Finally, the remaining ^1^H and ^13^C signals in **1** displayed marginal down- or up-field shifts from those of (*Z*)-5-(4-hydroxybenzylidene)imidazolidine-2,4-dione [10]. Accordingly, **1** was assigned as (*E*)-5-(4-hydroxybenzylidene)imidazolidine-2,4-dione and is reported as a new natural compound and named hemimycalin C.

**Figure 3:**
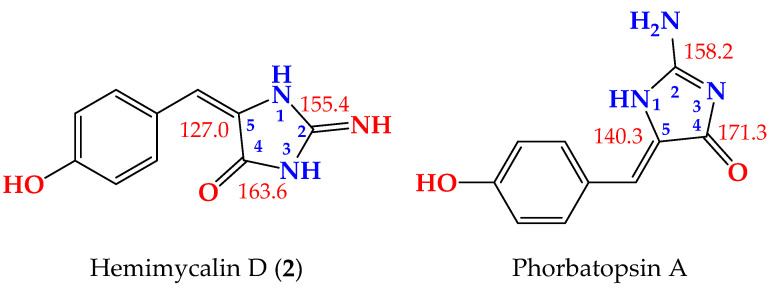
Comparison of the ^13^C NMR data (in DMSO-*d*_6_) of 2-iminoimidazolidin-4-one moiety in **2** (**left**) and 2-aminoimidazol-4-one moiety in phorbatopsin A (**right**).

Compound **2** (Figure 1) was obtained as a yellow powder with the molecular formula C_10_H_9_N_3_O_2_ obtained from the (+)-HRESIMS ion peak at *m*/*z* 204.0771 [M + H]^+^, being one atomic mass unit less than **1** and thus suggesting the replacement of one of the oxygen atoms in **2** with NH. The ^1^H (Figures S7 and S8) and ^13^C NMR (Figure S9) data of **2** (Table 3) were found to be in good agreement with those of **1** (Table 4). These data were supported also by HSQC (Figure S10) and HMBC (Figure S11) experiments. A comparison of the ^1^H and ^13^C NMR of **2** with those of **1** revealed marginal chemical shift differences between all NMR signals ranging from –0.39 to 0.0 ppm in the ^1^H NMR and from –0.1 to –1.8 ppm in the ^13^C NMR spectra (Table 3). A noticeable chemical shift difference was observed for C-2 (Δδ = –1.8 ppm) due to the replacement of the urea part (or hydantoin moiety) in **1** with a guanidine part (or 2-iminoimidazolidin-4-one) [25] in **2**. Additionally, to exclude the presence of 2-aminoimidazol-4-one moiety in **2**, the ^13^C NMR data of the 2-iminoimidazolidin-4-one moiety in **2** were compared with those reported for 2-aminoimidazol-4-one moiety, both measured in DMSO-*d*_6_ [23] (Figure 3). As shown in Figure 3, the ^13^C NMR data of 2-f in **2** were completely different from those of 2-aminoimidazol-4-one moiety in phorbatopsin A [23]. Furthermore, the HMBC correlations supported the assignment of the non-protonated carbons in **2** and the assignment of the 2-iminoimidazolidin-4-one moiety (Table 2 and Figure 2). Thus, **2** was assigned as (*E*)-5-(4-hydroxybenzylidene)-2-iminoimidazolidin-4-one and named hemimycalin D.

**Figure 4:**
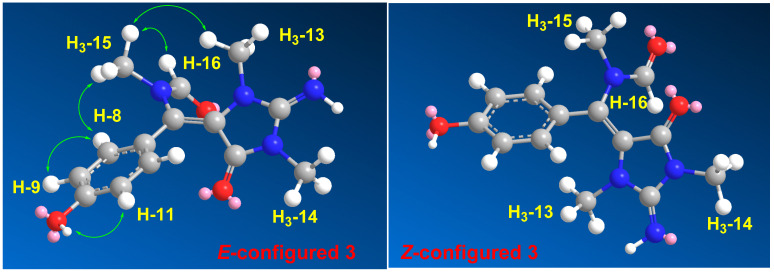
MM2-minimized energy drawings of **3** with observed NOESY correlations between H_3_-13 and H_3_-15 in the *E*-configured isomer.

Compound **3** (Figure 1) was found to possess the formula C_14_H_16_N_4_O_3,_ as shown by the (+)-HRESIMS ion peak at *m*/*z* 311.1118 for [M + Na]^+^. The ^1^H (Figures S12 and S13) and ^13^C NMR (Figure S14) spectra of **3** displayed typical resonances for a 1,4-substituted benzene ring, two *N*-methyls at δ_H/C_ 2.79/31.1 and 3.21/29.4 and an *N*-methylformamide at δ_H/C_ 2.83/33.6 (H_3_-15/C-15) and 7.91/166.3 (H-16/C-16). The ^1^H and ^13^C NMR data of **3** (Table 4) were found to be comparable with those reported for hemimycalin A [10], though featuring the replacement of the 1,3-dimethylimidazolidine-2,4-dione moiety in hemimycalin A [10] with 2-imino-1,3-dimethylimidazolidin-4-one moiety in **3**. This assignment was confirmed by HSQC (Figure S15) experiment and by HMBC (Figure S16) cross-peaks from H-12 (δ_H_ 7.48) to C-5 (qc, δ_C_ = 93.9), from H_3_-13 (δ_H_ 2.79) to C-2 (δ_C_ 153.6), and from H_3_-14 (δ_H_ 3.21) to C-2 and C-4 (δ_C_ = 149.7) (Table 5 and Figure 2). In addition, the placement of the *N*-methylformamide moiety at C-6 was confirmed by the HMBC cross peaks from H_3_-15 (δ_H_ =2.83) to C-6 (δ_C_ =126.1), from H_3_-15 to C-16 (δ_C_ = 166.3), and from H-16 (δ_H_ 7.91) to C-15 (δ_C_ = 33.6). The *E* configuration at the olefinic moiety Δ^5,6^ in **3** was confirmed from NOESY (Figure S17) correlations between H_3_-13 and H_3_-15, H_3_-15, and H-16, as well as between H_3_-15 and H-8,12. The NOESY correlations between H_3_-13 and H_3_-15 observed in the compound with *E* configuration at Δ^5,6^ were also confirmed by a comparison of the MM2-minimized drawings of the *E*-**3** against Z-**3** (Figure 4). It is very clear that the compound with the *E* configuration at Δ^5,6^ displayed significant NOESY between H_3_-13 and H_3_-15 (Table 4 and Figure 4). On the other hand, the isomer with the Z configuration at Δ^5,6^ was found to lack any correlation between these two methyl groups. Thus, the *E* configuration at Δ^5,6^ in **3** was confirmed. Accordingly, compound **3** was assigned as (*E*)-*N*-((4-hydroxyphenyl)(2-imino-1,3-dimethyl-5-oxoimidazolidin-4-ylidene)methyl)-*N*-methylformamide and named hemimycalin E.

An MTT assay showed **1**–**3** were mainly active against colorectal carcinoma (HCT 116) cells, with IC_50_ values of 8.6–18.8 μM (Table 5). On the contrary, **1**–**3** were moderately active towards triple-negative breast cancer (MDA-MB-231), with IC_50_ values of 21.5–31.7 μM, and inactive against human cervical carcinoma (Hela) cells. These data suggest that HCT 116 cells have higher sensitivity towards compound 3 than the other cell lines.

In a disk diffusion assay, **1**–**3** were evaluated for their effects on three pathogens at a concentration of 50 µg/disc. The compounds displayed high activities against *Candida albicans* (inhibition zones = 20–22 mm) and *Escherichia coli* (inhibition zones = 17–18 mm) but no effects on *Staphylococcus aureus* (Table 6). Finally, **1**–**3** displayed a minimum inhibitory concentration (MIC) value of 8 µM against *C. albicans* and *E. coli* (Table 6).

## 3. Materials and Methods

### 3.1. General Experimental Procedures

The IR spectra of **1**–**3** were recorded on a Shimadzu Infrared-400 spectrophotometer (Shimadzu, Kyoto, Japan). One- and two-dimensional NMR spectra were acquired on Bruker Avance DRX 600 MHz (Bruker, Rheinstetten, Germany) spectrometer. Positive ion HRESIMS data were obtained with a Micromass Q-ToF equipped with leucine enkephalin lock spray, using *m*/*z* 556.2771 [M + H]^+^ as a reference mass. Sephadex LH-20 (0.25–0.1 mm, Pharmacia) was used for column chromatography. Silica gel 60 F-254 plates (Merck) were used for TLC.

### 3.2. Biological Materials

**Figure 5:**
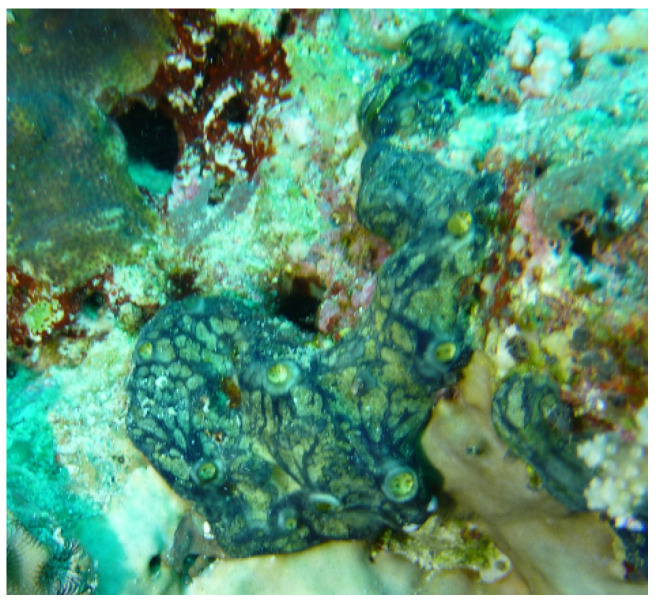
Underwater photograph of *Hemimycale* sp.

The sponge (Figure 5) was collected by hand using SCUBA at a depth of 13 m off Al-lith, Saudi Arabia. The dark blue encrusting sponge was found to be composed of a 1.5–2.0 cm thick soft mass. The skeleton of the sponge was plumose and composed of parallel loose bundles of thin spicules running from the substratum upwards through the sponge and fanning out at the surface. In between, there were many loose spicules. Bundles had a diameter of 30–50 µm and contained 12–20 spicules in cross-section. Siliceous spicules were straight and thin, either strongyles or styles but otherwise similar in shape and size, ranging from 215–255 × 2–4 µm. These details conformed with the description of the type specimen of the Red Sea sponge *Hemimycale arabica*, with which the current specimen was compared. A voucher specimen is kept in the Red Sea Invertebrates Collection at King Abdulaziz University under the code # DY21.

### 3.3. Purification of Compounds ***1***–***3***

The fresh sponge materials (430 g) were crushed into small pieces and macerated in MeOH (3 × 1500 mL), and the concentrated methanolic extract was chromatographed on Sephadex LH-20 (150 g) with MeOH–CH_2_Cl_2_ (1:1). The cytotoxic fraction (320 mg) was subjected to Sep-Pak C18 Cartridge (Waters, 10 g) using H_2_O–MeOH gradients to provide five major fractions. The fraction eluted with 60% MeOH (86 mg) was purified by HPLC (Cosmosil, 250 × 10 mm) using 30% CH_3_CN to afford **1** (7.0 mg) and **2** (3.2 mg). Furthermore, the fraction eluted with 70% MeOH (34 mg) was purified by HPLC (Cosmosil, 250 × 10 mm) to afford **3** (4.1 mg).

### 3.4. Spectral Data of the Compounds

(1) Hemimycalin C (**1**). Yellow powder; IR γ_max_ (film) 3382, 1721, 1644, 1595 cm^−1^; NMR data: see Table 1 and Table 2; HRESIMS *m/z* 205.0609 (calculated for C_10_H_9_N_2_O_3_ [M + H]^+^, 205.0607).
(2) Hemimycalin D (**2**). Yellow powder; IR γ_max_ (film) 3374, 1724, 1646, 1594 cm^−1^; NMR data: see Table 3 and Table 4; HRESIMS *m/z* 204.0771 (calculated for C_10_H_10_N_3_O_2_ [M + H]^+^, 204.0767).
(3) Hemimycalin E (**3**) Yellow powder; IR γ_max_ (film) 3375, 1723, 1647, 1595 cm^−1^; NMR data: see Table 5; HRESIMS *m/z* 311.1118 (calculated for C_14_H_16_N_4_O_3_Na [M + Na]^+^, 311.1114).

### 3.5. Biological Evaluation of the Compounds

#### 3.5.1. Cytotoxicity of the Compounds

##### Culture of Cell Lines

HCT116 (Colorectal carcinoma, ATCC CCL-247) and HeLa (human cervical carcinoma, ATCC CCL-2) cells were cultured in an RPMI 1640 medium with 10% FBS, and 1% penicillin–streptomycin, while MDA-MB-231 cells (triple-negative breast cancer, ATCC HTB-26) were cultured in a DMEM medium with 1% penicillin–streptomycin and 10% FBS.

##### Evaluation of Antiproliferative Activity

The evaluation of the antiproliferative effects of **1**–**3** was performed using an MTT, assay as reported earlier [26,27]. The cells were incubated at 37 °C overnight in 5% CO_2_/air. After that, the compounds were added to the top row of a 96-well microtiter plate, and descendant serial dilutions (1:4) of the concentration were performed followed via the incubation of the cells with the compounds for 72 h. Using the CellTiter 96 AQueous non-radioactive cell proliferation protocol, the cells’ viability was estimated at 490 nm on a Molecular Devices Emax microplate reader. The IC_50_ values of the compounds (expressed in μM) were determined using the program SOFTmax PRO. 5-Flourouracil and DMSO were used as positive and negative controls, respectively. A concentration of 25 μM was set as a cutoff value in this assay.

#### 3.5.2. Disk Diffusion Assay

The antimicrobial effects of **1**–**3** were evaluated using a disc diffusion assay at 50 µg/disc against *E. coli* (ATCC 25922), *C. albicans* (ATCC 14053), and *S. aureus* (ATCC 25923), as described previously [28,29,30]. Ciprofloxacin and ketoconazole served as positive controls in the antimicrobial assay, while DMSO was used as a negative control.

#### 3.5.3. Evaluation of the MIC Values

The determination of the MIC values of **1**–**3** against *C. albicans* and *E. coli* was performed using a macro-dilution assay, as previously reported [31].

## 4. Conclusions

The bioassay-directed partition and purification of the cytotoxic fraction of the Red Sea sponge *Hemimycale* sp. provided three new alkaloids: hemimycalins C–E (**1**–**3**). The structures of the compounds were assigned via analyses of their spectral data. Interestingly, hemimycalin C (**1**) was found to possess an *E* configuration [25] at Δ^5,6^ instead of the previously reported *Z* configuration of Δ^5,6^. In addition, hemimycalins D and E (**2** and **3**) were found to possess the 2-iminoimidazolidin-4-one [25] backbone instead of hydantoin (imidazolidine-2,4-dione) moiety in previously reported alkaloids from the genus *Hemimycale*. Furthermore, hemimycalin D (**2**) was found to share the *E* configuration at Δ^5,6^ with hemimycalin C (**1**). Consequently, the *E*-configured **1** and **2** were shown to possess higher chemical shift values for C-6 than the Z-configured compounds, while H-6 [23,24,25] in the *E*-configured compounds [23,24,25] was found to resonate at lower chemical shift values than in the *Z*-configured ones.

Hemimycalins C–E showed significant cytotoxic effects and selective antimicrobial effects against *E. coli* and *C. albicans*, making them potential scaffolds for the development of drug leads.

The current findings provide a deeper insight and understanding of the chemical diversity and biological activities of the secondary metabolites of the Red Sea sponge *Hemimycale* sp.

## Figures and Tables

**Table 1:** NMR data of **1** (600 MHz for ^1^H and 150 for ^13^C, DMSO-*d*_6_).

Position	δ_C_, Type	δ_H_ (Mult., *J* in Hz)	HMBC
2	153.6, C		
4	163.6, C		
5	127.0, C		
6	116.8, CH	6.23 (s)	C-4, C-8, C-12
7	124.1, C		
8	131.8, CH	7.82 (d, 9.0)	C-6, C-7, C-10, C-12
9	115.0, CH	6.72 (d, 9.0)	C-7, C-10, C-11
10	158.0, C		
11	115.0, CH	6.72 (d, 9.0)	C-7, C-10, C-9
12	131.8, CH	7.82 (d, 9.0)	C-6, C-7, C-10, C-8
NH, OH		10.50 (br hump)	

**Table 2:** Comparison of ^13^C NMR data between (*E*)-**1** and (*Z*)-**1** (DMSO-*d*_6_).

Position	(*E*)-1		(*Z*)-1 ^a^		Δδ (*E*−*Z*) in ppm	
	δ_C_, Type	δ_H_, (Mult., *J* in Hz)	δ_C_, Type	δ_H_, (Mult., *J* in Hz)	Δδ_C(*E*−*Z*)_	Δδ_H(*E*−*Z*)_
2	153.6, C		155.7, C		−2.1	
4	163.6, C		165.7, C		−2.1	
5	127.0, C		125.3, C		+1.7	
6	116.8, CH	6.23 (s)	109.2, CH	6.33 (s)	+7.6	−0.10
7	124.1, C		123.8, C		+0.3	
8	131.8, CH	7.82 (d, 9.0)	131.2, CH	7.46 (d, 9.0)	+0.6	+0.36
9	115.0, CH	6.72 (d, 9.0)	115.6, CH	6.76 (d, 9.0)	−0.6	−0.04
10	158.0, C		158.0, C		0.0	
11	115.0, CH	6.72 (d, 9.0)	115.6, CH	6.76 (d, 9.0)	−0.6	−0.04
12	131.8, CH	7.82 (d, 9.0)	131.2, CH	7.46 (d, 9.0)	+0.6	+0.36

^a^ Data from reference [10].

**Table 3:** NMR data of **2** (600 MHz for ^1^H and 150 for ^13^C, DMSO-*d*_6_).

Position	δ_C_, Type	δ_H_ (Mult., *J* in Hz)	HMBC
2	155.4, C		
4	163.2, C		
5	126.5, C		
6	116.5, CH	6.23 (s)	C-4, C-8, C-12
7	123.5, C		
8	131.6, CH	7.43 (d, 9.0)	C-6, C-7, C-10, C-12
9	114.8, CH	6.72 (d, 9.0)	C-7, C-10, C-11
10	157.9, C		
11	114.8, CH	6.72 (d, 9.0)	C-7, C-10, C-9
12	131.6, CH	7.43 (d, 9.0)	C-6, C-7, C-10, C-8
NH, OH		10.50 (br hump)	

**Table 4:** NMR data of **3** (600 MHz for ^1^H and 150 for ^13^C, DMSO-*d*_6_).

Position	δ_C_, Type	δ_H_ (Mult., *J* in Hz)	HMBC	NOESY
2	153.6, C			
4	149.7, C			
5	93.9, C			
6	126.1, C			
7	124.5, C			
8	131.3, CH	7.48 (d, 8.4)	C-6, C-7, C-10	H-9, OH, H_3_-15
9	115.2, CH	6.73 (d, 8.4)	C-7, C-10	H-8
10	159.8, C			
OH		10.78 (brs)	C-10	H-9, H-11
11	115.2, CH	6.73 (d, 8.4)	C-7, C-10	H-12, OH
12	131.3, CH	7.48 (d, 8.4)	C-6, C-7, C-10	H-11, H_3_-15
13	31.1, CH_3_	2.79 (s)	C-2, C-5	H_3_-15
14	29.4, CH_3_	3.21 (s)	C-2, C-4	
15	33.6, CH_3_	2.83 (s)	C-6, C-16	H-8, H-12, H-16, H_3_-13
16	166.3, CH	7.91 (s)	C-15	H_3_-15

**Table 5:** Antiproliferative effects of **1**–**3**.

Compound	IC_50_ (μM) (Mean + SEM) ^a^		
	MDA-MB-231	HeLa	HCT 116
1	28.5 ± 0.21	≥25.0	18.6 ± 0.12
2	31.7 ± 0.25	≥25.0	17.1 ± 0.09
3	21.5 ± 0.18	≥25.0	8.6 ± 0.06
5-FU ^b^	13.0 ± 0.30	12.3 ± 0.25	4.6 ± 0.23

^a^ The results are the mean of three independent experiments; ^b^ 5-Flourourcail, a positive drug.

**Table 6:** Antimicrobial activities of **1**–**3**.

Compound	Inhibition Zones (mm) and MIC Values (µM)				
	*C. albicans*	MIC (µM)	*E. coli*	MIC (µM)	*S. aureus*
1	22	8	17	8	NI
2	20	8	18	8	NI
3	20	8	17	8	NI
Ciprofloxacin ^a^	NT		30	0.08	22
Ketoconazole ^b^	30	0.26	NT		NT

^a^ Positive antibacterial control (5 μg/disc); ^b^ positive antifungal control (50 μg/disc); NI = no inhibition; NT = not tested.

## Data Availability

Data is contained within the article or Supplementary Material.

## Supplementary Materials

The following are available online at https://www.mdpi.com/article/10.3390/md19120691/s1, Figures S1–S19: ^1^HNMR, ^13^C NMR, DEPT, COSY, HSQC, HMBC, and NOESY spectra of hemimycalins C–E (**1**–**3**).
