# Supplementary material for: Hemimycalins C–E; Cytotoxic and Antimicrobial Alkaloids with Hydantoin and 2-Iminoimidazolidin-4-one Backbones from the Red Sea Marine Sponge Hemimycale sp
Source: Mar Drugs. 2021 Dec 2;19(12):691. doi: 10.3390/md19120691 (PMC8705819; doi:10.3390/md19120691)
Supplement: Supplementary file 1 [file marinedrugs-19-00691-s001.zip › marinedrugs-1476130-supplementary.pdf]

Supporting Information

Figure S1. ^{1}H NMR spectrum of hemimycalin C (**1**) ······ 1
Figure S2. Expansion of ^{1}H NMR spectrum of hemimycalin C (**1**) ······ 1
Figure S3. ^{13}C NMR spectrum of hemimycalin C (**1**) ······ 2
Figure S4. DEPT spectrum of hemimycalin C (**1**) ······ 2
Figure S5. Multiplicity-edited HSQC spectrum of hemimycalin C (**1**) ······ 3
Figure S6. ^{1}H-^{13}C HMBC spectrum of hemimycalin C (**1**) ······ 4
Figure S7. ^{1}H NMR spectrum of hemimycalin D (**2**) ······ 5
Figure S8. Expansion of ^{1}H NMR spectrum of hemimycalin D (**2**) ······ 5
Figure S9. ^{13}C NMR spectrum of hemimycalin D (**2**) ······ 6
Figure S10. Multiplicity-edited HSQC spectrum of hemimycalin D (**2**) ······ 7
Figure S11. ^{1}H-^{13}C HMBC spectrum of hemimycalin D (**2**) ······ 8
Figure S12. ^{1}H NMR spectrum of hemimycalin E (**3**) ······ 9
Figure S13. Expansion of ^{1}H NMR spectrum of hemimycalin E (**3**) ······ 9
Figure S14. ^{13}C NMR spectrum of hemimycalin E (**3**) ······ 10
Figure S15. Multiplicity-edited HSQC spectrum of hemimycalin E (**3**) ······ 11
Figure S16. ^{1}H-^{13}C HMBC spectrum of hemimycalin E (**3**) ······ 12
Figure S17. ^{1}H-^{1}H NOESY spectrum of hemimycalin E (**3**) ······ 13
Figure S18. Dose response curves of compounds **1-3** against MDA-MB-231 ······ 14
Figure S19. Dose response curves of compounds **1-3** against HCT116 ······ 15

Figure S1. ^{1}H NMR spectrum of hemimycalin C (**1**) (DMSO-*d₆*).

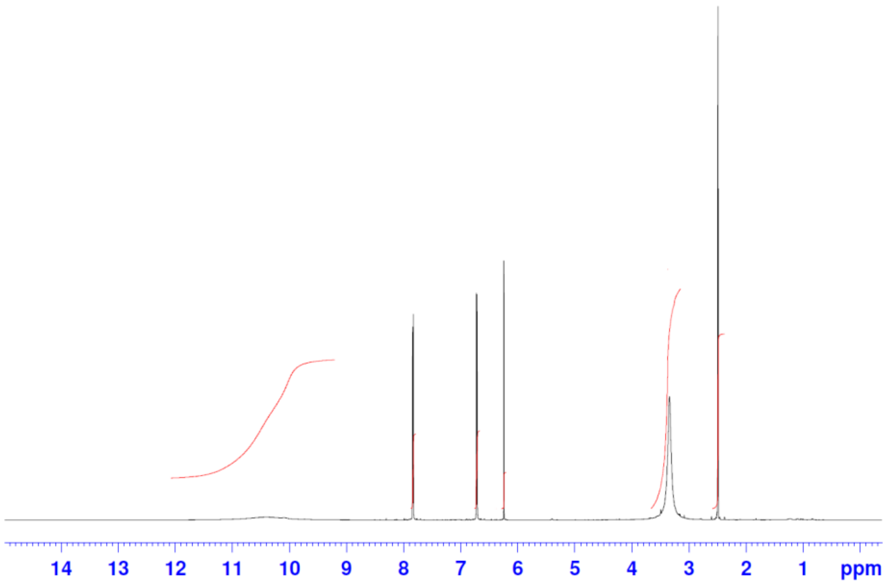

Figure S2. Expansion of ^{1}H NMR spectrum of hemimycalin C (**1**) (DMSO-*d₆*).

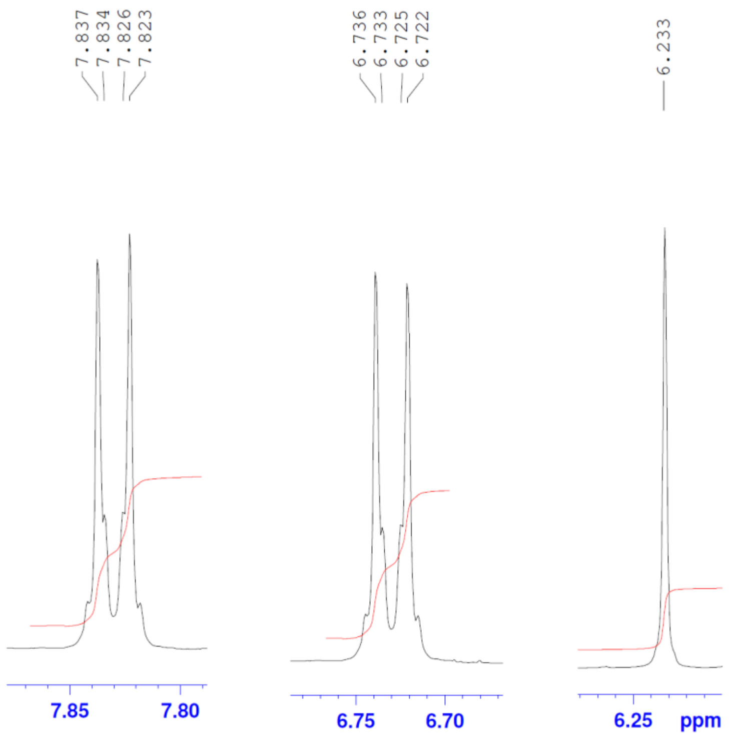

Figure S3. ^{13}C NMR spectrum of hemimycalin C (**1**) (DMSO-d_6).

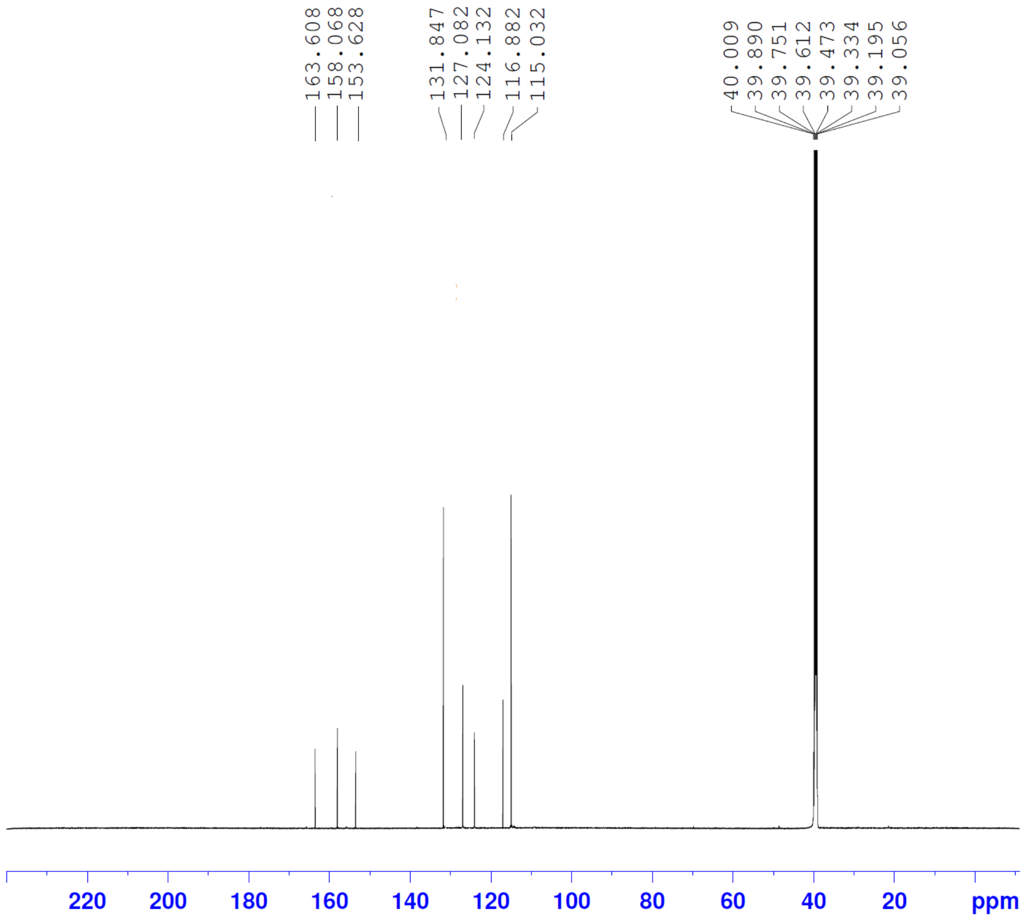

Figure S4. DEPT spectrum of hemimycalin C (**1**) (DMSO-d_6).

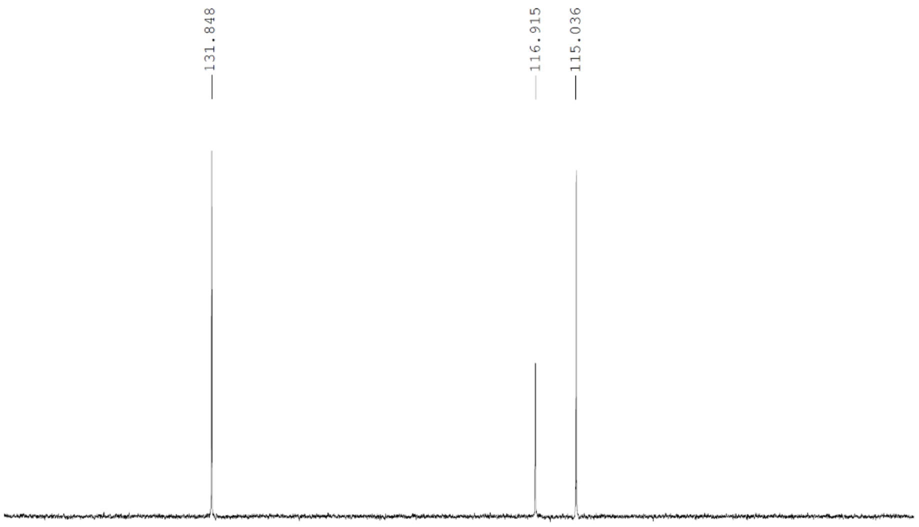

Figure S5. Multiplicity-edited HSQC spectrum of hemimycalin C (**1**) (DMSO-d_6).

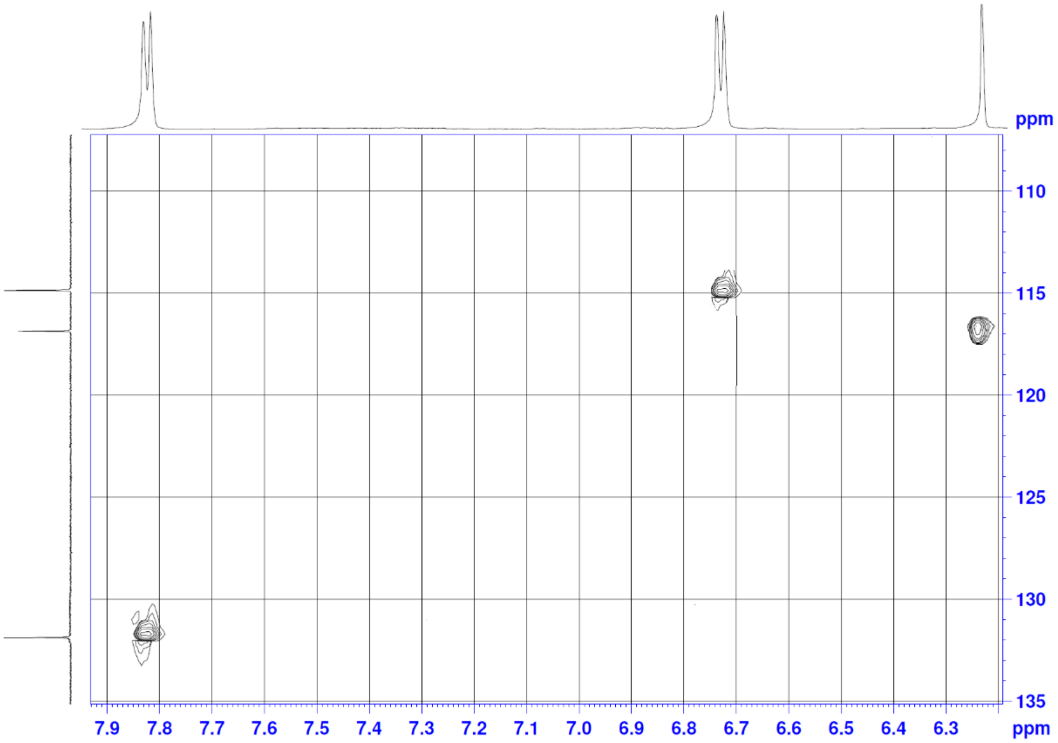

Figure S6. ^{1}H-^{13}C HMBC spectrum of hemimycalin C (**1**) (DMSO-d_6).

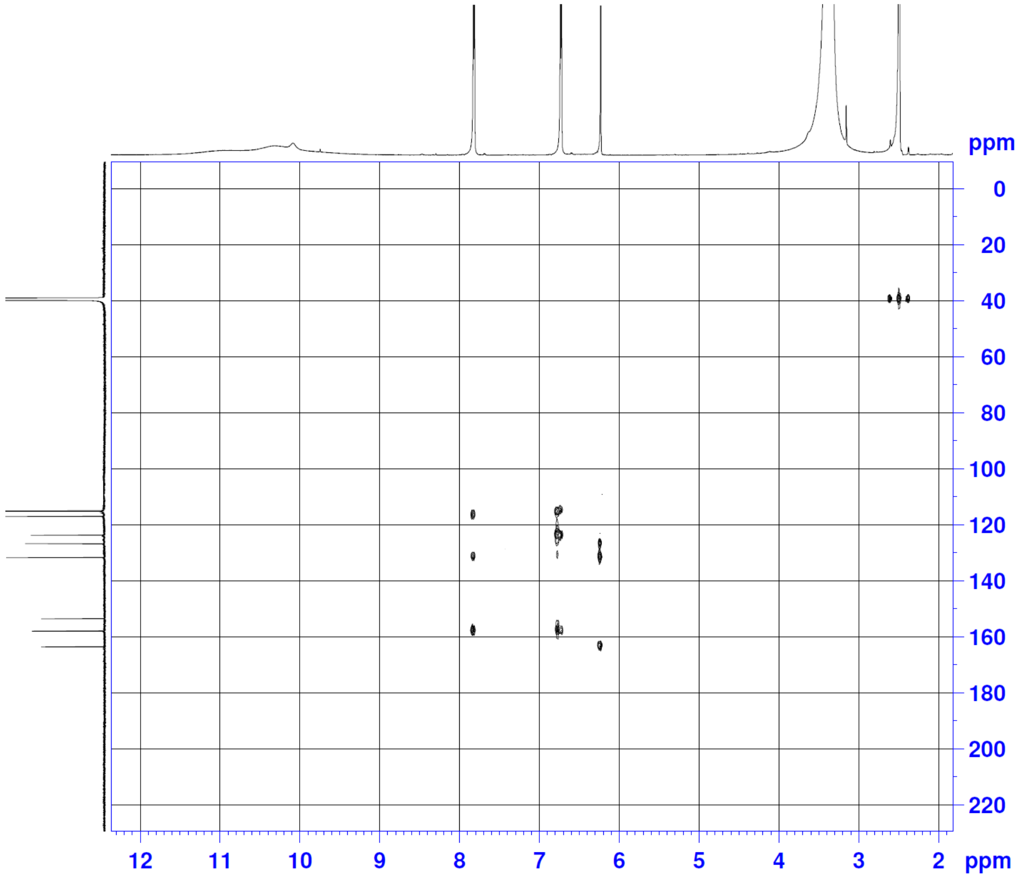

Figure S7. ^{1}H NMR spectrum of hemimycalin D (**2**) (DMSO-d_6).

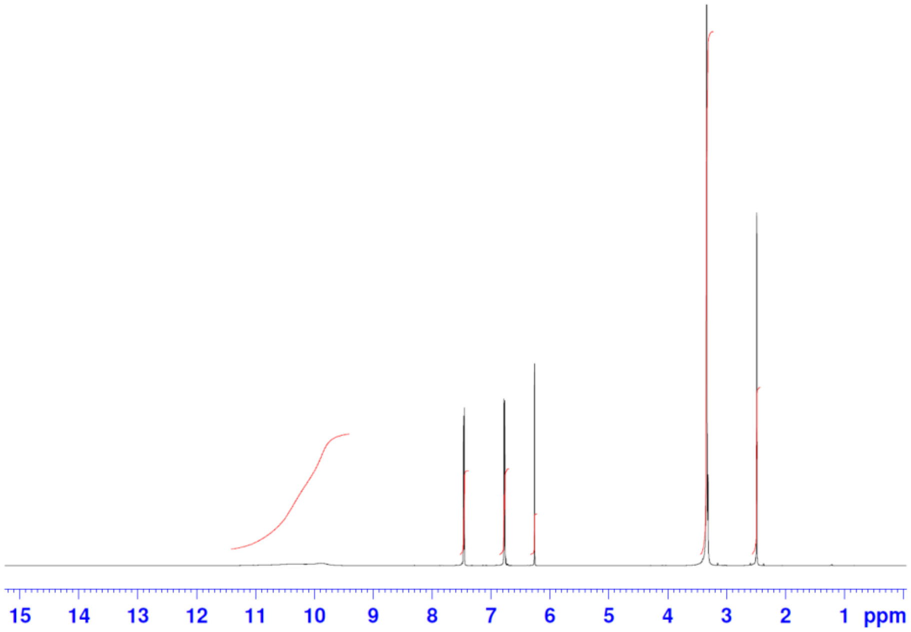

Figure S8. Expansion of ^{1}H NMR spectrum of hemimycalin D (**2**) (DMSO-d_6).

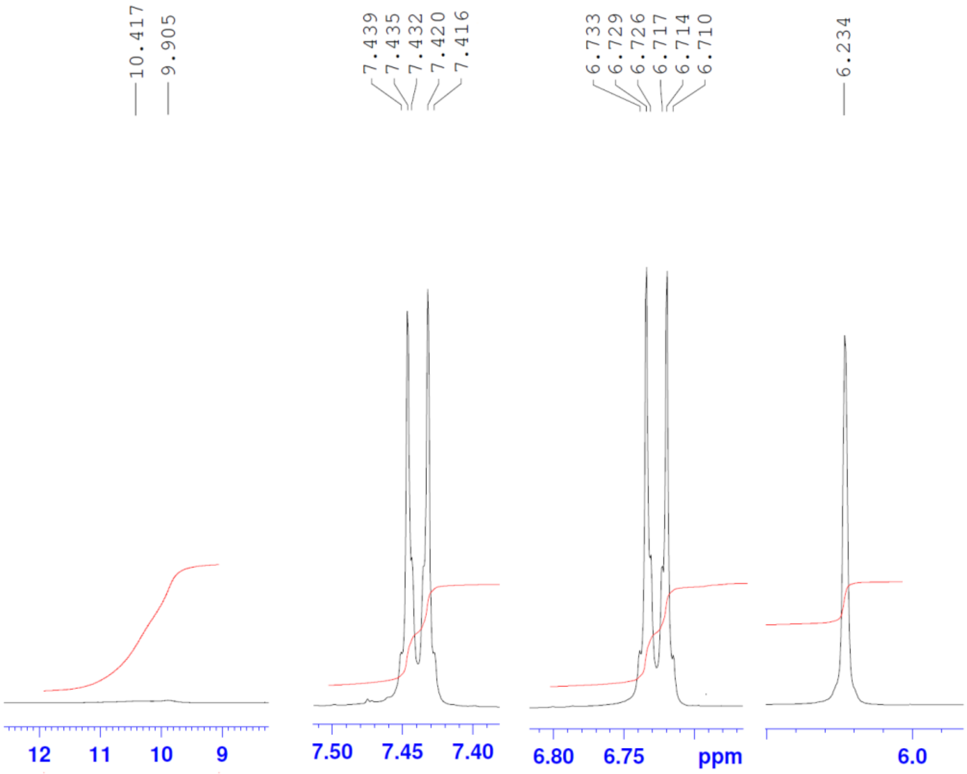

Figure S9. ^{13}C NMR spectrum of hemimycalin D (**2**) (DMSO-d_6).

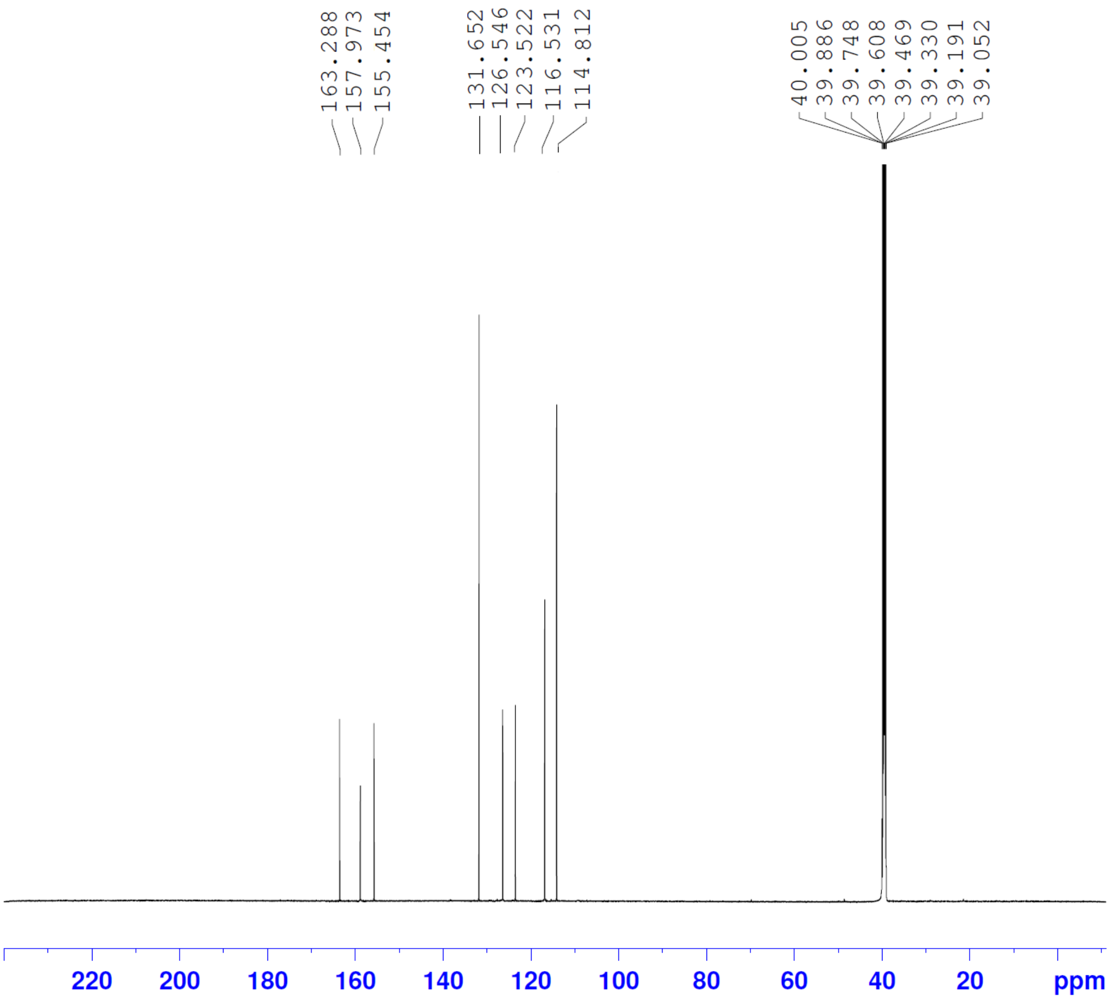

Figure S10. Multiplicity-edited HSQC spectrum of hemimycalin D (**2**) (DMSO-d_6).

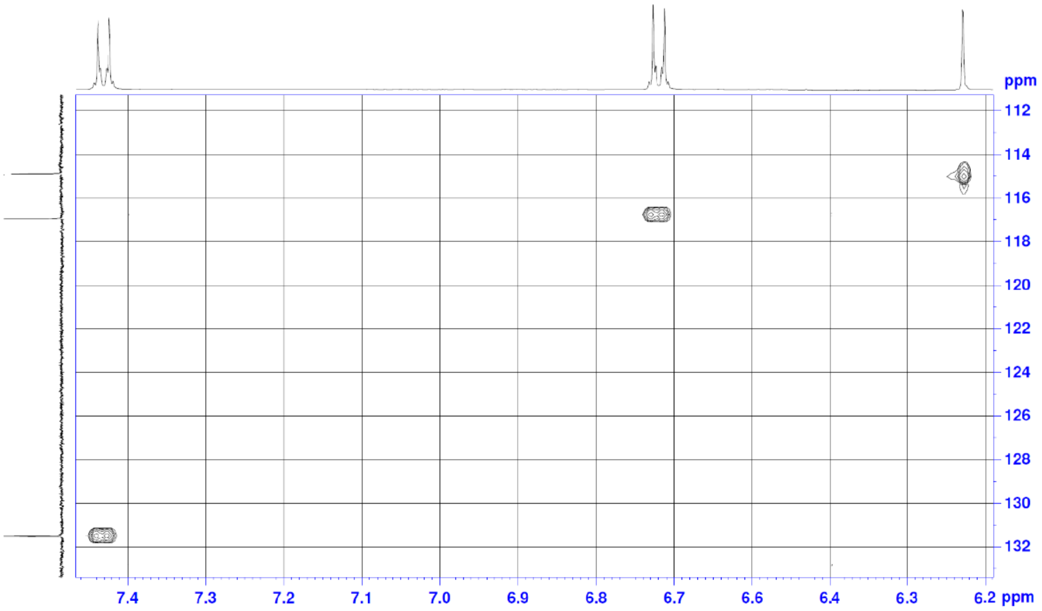

Figure S11. ^{1}H-^{13}C HMBC spectrum of hemimycalin D (**2**) (DMSO-d_6).

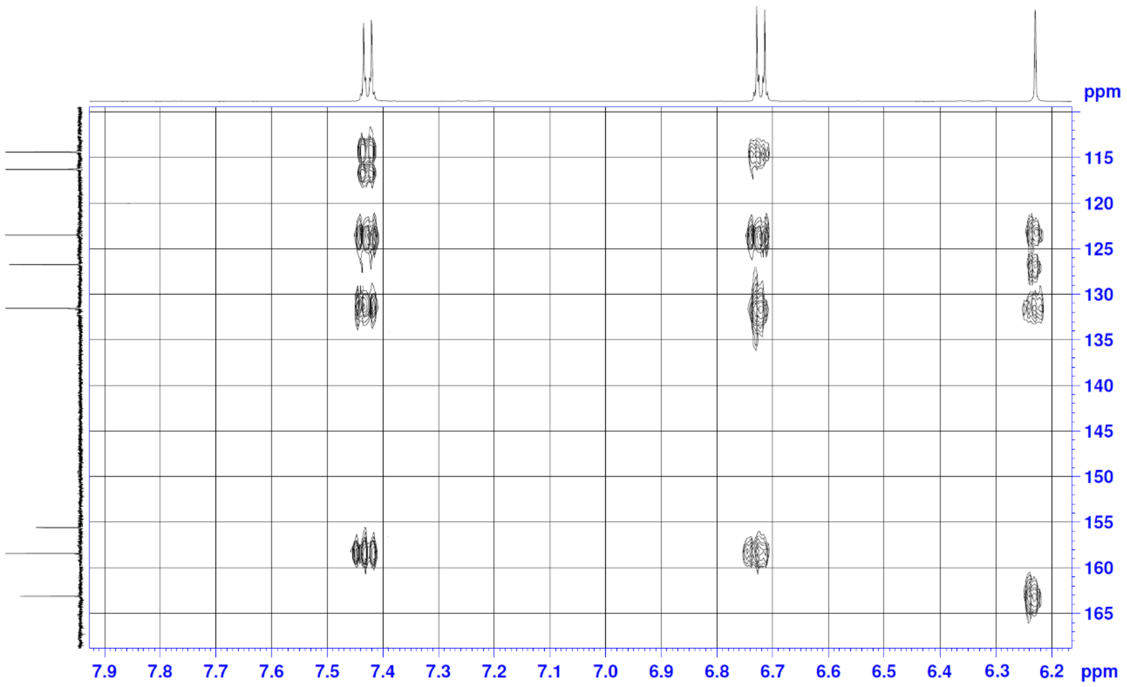

Figure S12. ^{1}H NMR spectrum of hemimycalin E (**3**) (DMSO-d_6).

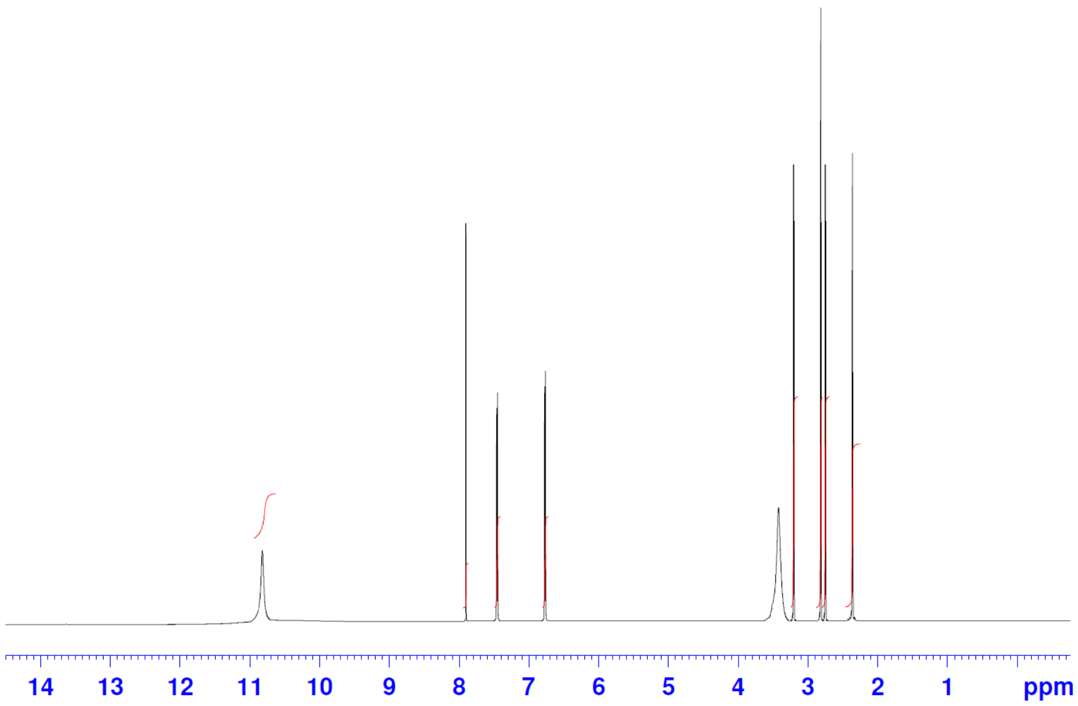

Figure S13 Expansion of ^{1}H NMR spectrum of hemimycalin E (**3**) (DMSO-d_6).

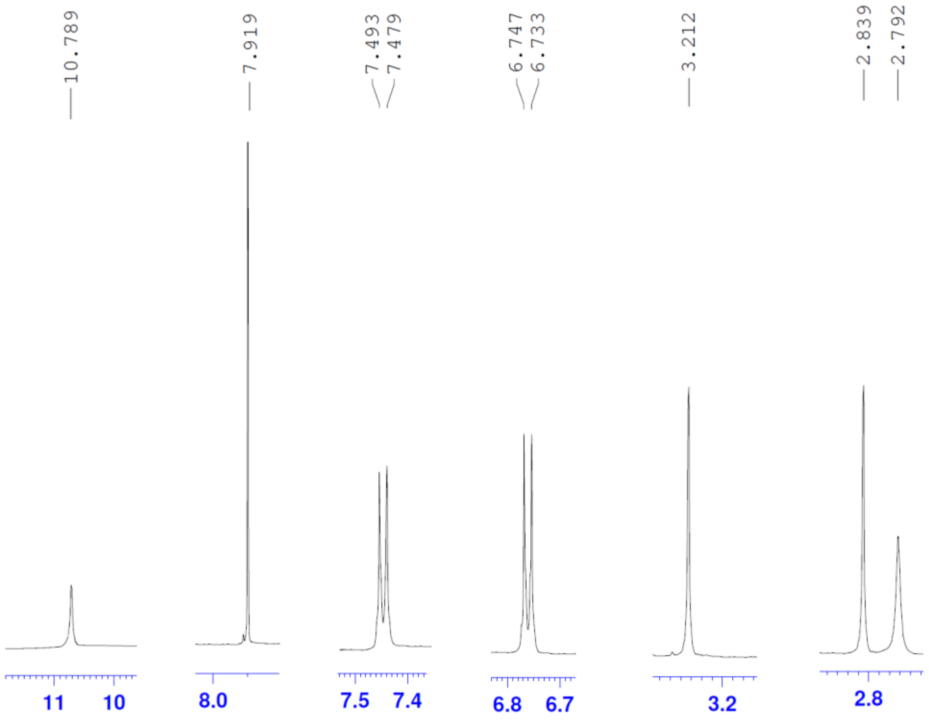

Figure S14. ^{13}C NMR spectrum of hemimycalin E (**3**) (DMSO-d_6).

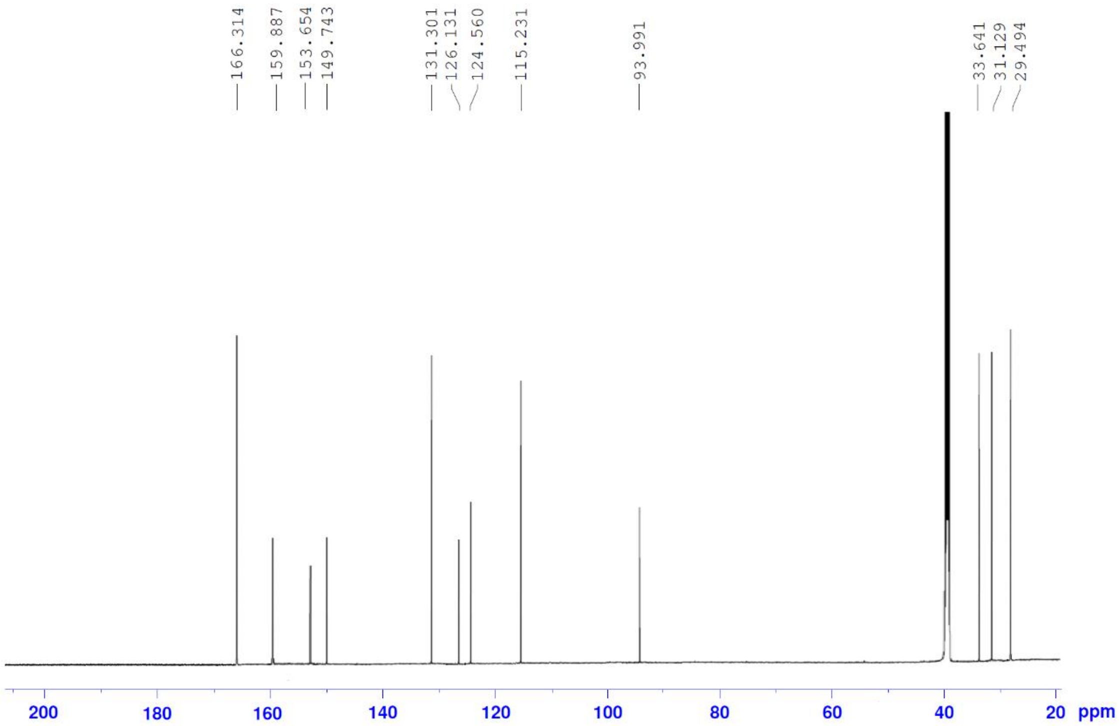

Figure S15. Multiplicity-edited HSQC spectrum of hemimycalin E (**3**) (DMSO-d_6).

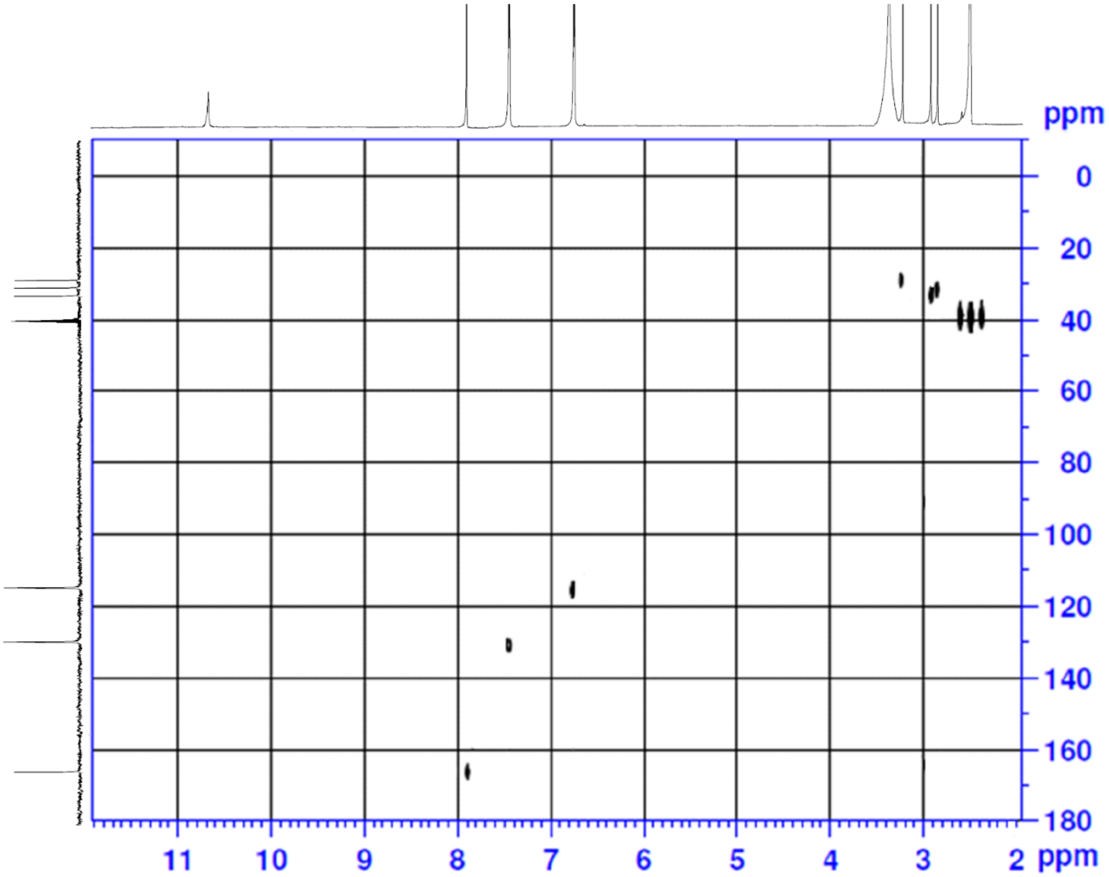

Figure S16. ^{1}H-^{13}C HMBC spectrum of hemimycalin E (**3**) (DMSO-d_6).

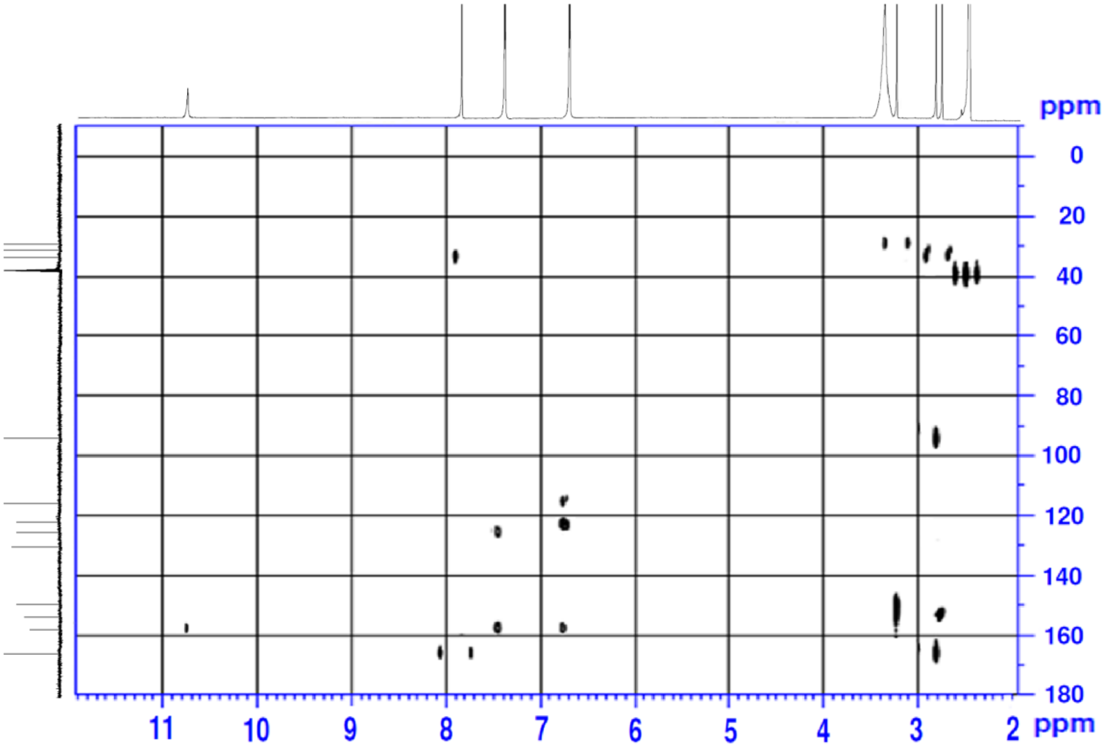

Figure S17. ^{1}H-^{13}H NOESY spectrum of hemimycalin E (**3**) (DMSO-d_6).

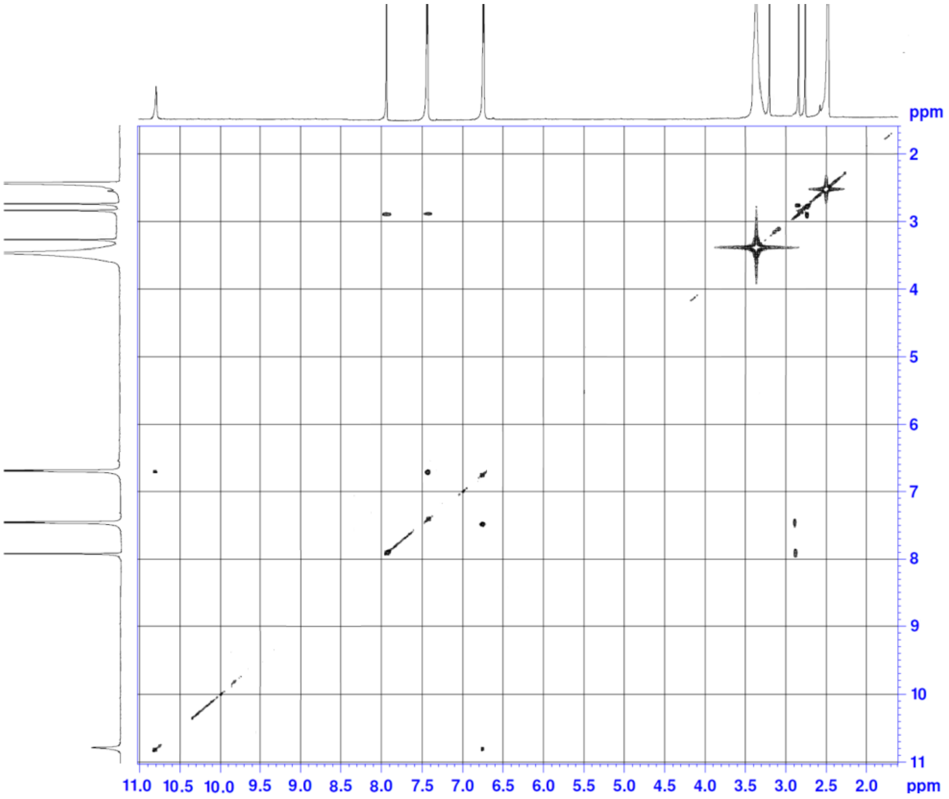

Figure S18. Dose-response curves of compounds **1-3** against MDA-MB-231.

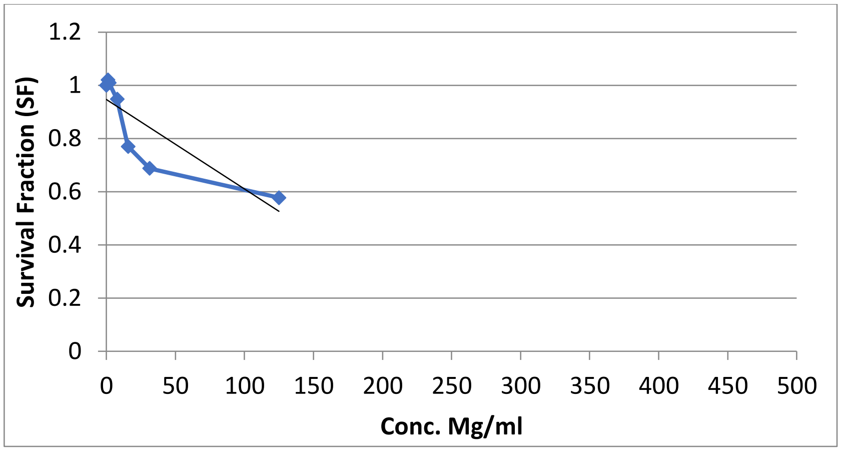

Compound **1** against MDA-MB-231

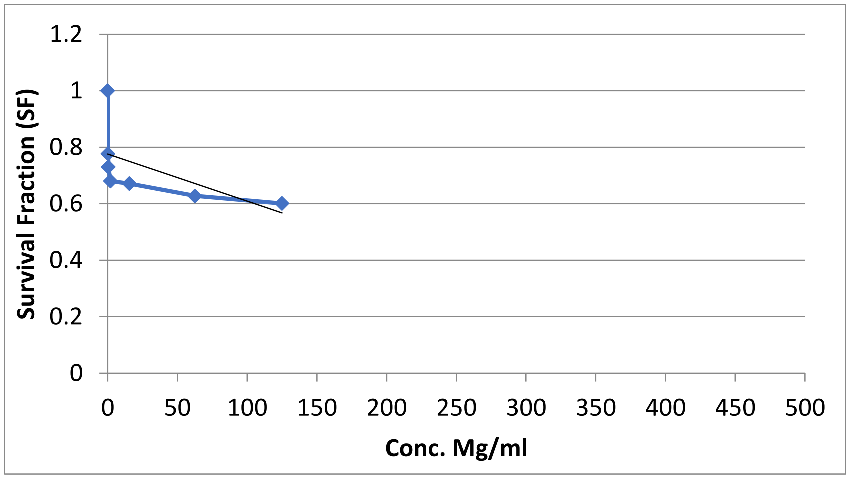

Compound **2** against MDA-MB-231

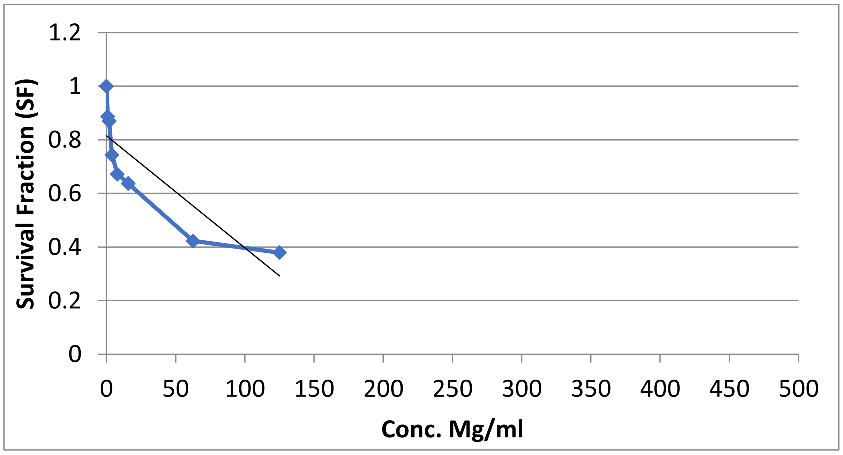

Compound **3** against MDA-MB-231

Figure S19. Dose-response curves of compounds **1-3** against HC T116.

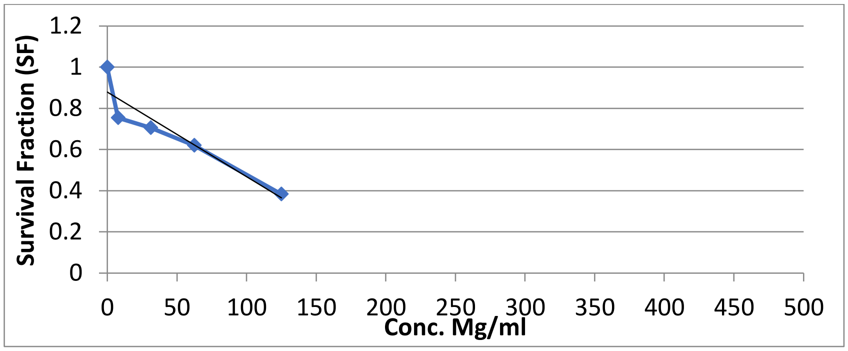

Compound **1** against HCT 116

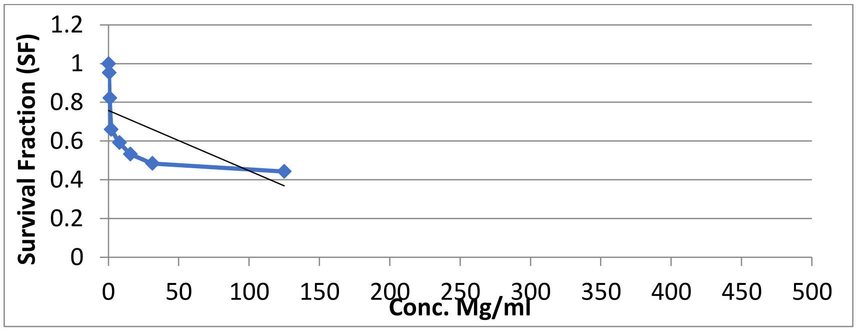

Compound **2** against HCT 116

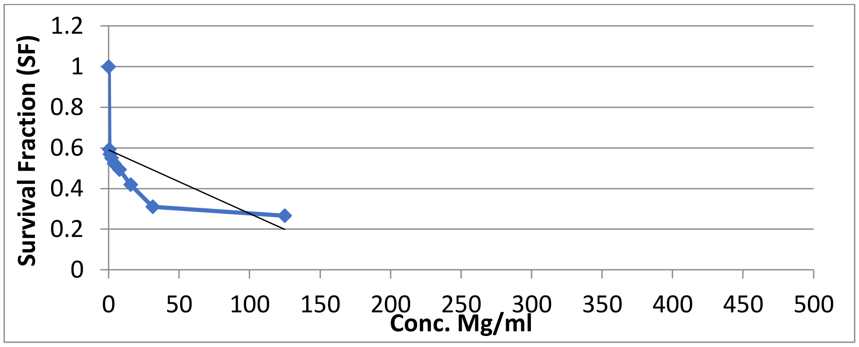

Compound **3** against HCT 116
